# Discriminant Validity of the WAIS-R Digit Symbol Substitution Test in Subjective Cognitive Decline, Mild Cognitive Impairment (Amnestic Subtype) and Alzheimer’s Disease Dementia (ADD) in Greece

**DOI:** 10.3390/brainsci11070881

**Published:** 2021-06-30

**Authors:** Marianna Tsatali, Eleni Poptsi, Despina Moraitou, Christina Agogiatou, Evaggelia Bakoglidou, Moses Gialaouzidis, Chrysa Papasozomenou, Aikaterini Soumpourou, Magdalini Tsolaki

**Affiliations:** 1 Department of Psychology, Greek Association of Alzheimer Disease and Related Disorders (GAADRD), 54643 Thessaloniki, Greece; poptsielena@gmail.com (E.P.); despoinamorait@gmail.com (D.M.); ag.christina@gmail.com (C.A.); litsa.bak@gmail.com (E.B.); moses_gf@hotmail.com (M.G.); chrysa.kav@gmail.com (C.P.); katerinasoum@gmail.com (A.S.); tsolakim1@gmail.com (M.T.); 2 Laboratory of Psychology, Section of Cognitive and Experimental Psychology, School of Psychology, Aristotle University of Thessaloniki (AUTh), 54124 Thessaloniki, Greece; 3 Center for Interdisciplinary Research and Innovation, Laboratory of Neurodegenerative Diseases, Aristotle University of Thessaloniki (CIRI-AUTh), 57001 Thessaloniki, Greece

**Keywords:** digit symbol substitution test (DSST), WAIS-R, validation study, cut-off scores, psychometric tools, neuropsychology, aMCI, Alzheimer’s disease dementia (ADD)

## Abstract

Objective: The aim of the current study was to estimate the discriminant potential and validity of the Digit Symbol Substitution Test (DSST) of the WAIS-R in the Greek elderly population meeting criteria for subjective cognitive decline (SCD), mild cognitive impairment (aMCI; amnestic subtype), or Alzheimer’s disease dementia (ADD). Method: Four hundred eighty-eight community-dwelling older adults, visitors of the Day Center of Alzheimer Hellas, participated in the study. Two hundred forty-three of them met the criteria for ADD, one hundred eighty-two for aMCI and sixty-three for SCD. Results: Path analysis indicated that the DSST score is affected by age group, educational level, and diagnostic category, but is not affected by gender. The ROC curve analysis showed that the DSST sum score could perfectly differentiate SCD from ADD patients, whereas test’s discriminant potential between aMCI and dementia ADD’s subtype was satisfactory. However, DSST was unable to separate the SCD from the aMCI group. Conclusion: It appears that the DSST is unable to separate the SCD from aMCI population. Therefore, the test in question may be insensitive to incipient cognitive decline. On the contrary, the discriminant potential of the DSST as regards SCD and ADD is excellent, while discrimination between aMCI and ADD is good.

## 1. Introduction

The Digit Symbol Substitution Test (DSST) has been a prominent subtest measuring cognitive performance in every version of the Wechsler Intelligence scale since its initial publication [1,2]. The DSST WAIS-R constitutes a paper-and-pencil cognitive test, which requires matching symbols with their corresponding numbers over a 90-s time interval.

Given the fact that it is widely used as part of the neuropsychological evaluation in older adult populations, its use is highly recommended both in research and also clinical practice in this population. The DSM-V criteria [3] classifies two different types of neurocognitive disorders, that is, minor and major neurocognitive disorders, also referred to as mild cognitive impairment (MCI) and dementia, respectively. MCI is perceived as a clinical entity prior to dementia and therefore it is of great significance to distinguish those older adults with MCI from those with subjective cognitive decline (SCD), which is a very early stage of cognitive impairment in which memory complaints but not objective deficits exist. Νevertheless, Zygouris et al. [4] found that Virtual Supermarket Test (VST) could satisfactorily discriminate MCI patients from those with SCD, as compared to MMSE and MoCA screening tests, whereas Poptsi et al. [5] suggest that the REflexes MEasurement DEviceS for Alzheimer battery (REMEDES4Alz) adequately distinguishes SCD from MCI. On the other hand, Petrazzuoli et al. [6] support that neither single nor combinations of tests, such as the clock-drawing test (CDT), the test of attention/executive function (AQT), as well as the Mini Mental State Examination (MMSE) could differentiate MCI from SCD with adequately high accuracy. To sum up, conducting relevant studies in order to classify the aforementioned groups is quite crucial in research, but mainly in developing structured non-pharmacological interventions to boost their cognitive function. At first, the DSST was assumed to measure learning ability (pairing and free recall) [7], with the individual being called upon to learn a number of digits and their respective symbols in order to perform the maximum possible correct combinations during the time allotted. However, other studies [8,9] argued that the Digit Symbol evaluates processing speed rather than learning and memory functions. Taking into account the equivocal role of the previous studies [10], while highlighting processing speed as a significant feature of the Digit Symbol-WAIS-R, nonetheless supported that short-term memory capacity and learning processes are primarily measured by this test. In our study in particular, which included participants without motor disorders and comprised only of older adults and no adults in younger age groups, the DSST was used to measure incidental learning.

It is worth mentioning that the DSST WAIS-R differs from its counterpart included in the WAIS-IV. More specifically, the pairings of numbers and symbols differ between the two versions, as the combinations between the pairings appear with equal frequency per each row in the WAIS-IV. Another difference between the two versions refers to the fact that the newest one does not involve the memory component, contrarily to the WAIS-IV. In every version, the Digit Symbol, named Coding in the WAIS-IV, has constituted an inseparable part of the Wechsler intelligence scale since its first publication [2]. Moreover, the memory component measured by the DSST WAIS-R but not by the DSST WAIS-IV is primarily impaired in neurodegenerative diseases; consequently, this version of the DSST could be proven more useful in detecting older adults with objective memory deficits and distinguishing them from their cognitively intact peers and/or those with no obvious cognitive impairment. Specifically, the later versions of the DSST called Coding focus primarily on processing speed, and therefore its use is not suggested in older adults.

The DSST constitutes an integral part of the neuropsychological assessment in older adults as one of the most widely administered tools in clinical neuropsychology [11]. Initially, one main advantage of the DSST refers to its easy and quick administration, with a rather insignificant language, cultural and educational impact (Jaeger, 2018). According to the meta-analysis by Hoyer, Stawski, Wasylyshyn and Verhaeghen [12], in contrast to educational level, age appears to be strongly related to the DSST performance [13], and therefore its use in the elderly population is highly recommended on a routine basis. In contrast, Kreiner and Ryan [7] reported that, based on empirical evidence, age decline in the DSST could primarily reflect motor abilities rather than the mnemonic demands of the test. This is further supported by the study by Erber, Botwinick and Storandt [14], who showed that even when older adults were allowed to learn the digit–symbol pairs, their DSST scores did not meliorate. However, Kreiner and Ryan [7] reported that these results may be explained by the fact that older adults used a conservative strategy of finding the symbol for each number instead of using the newly learned pairs.

Secondly, the DSST records the lowest correlation levels with the IQ performance as compared to other WAIS subtests, since it is negatively associated to age [15]. Therefore, the possibility of attributing participants’ performances to their IQ scores can be safely ruled out. Thirdly, Lezak [16] supported that among the other WAIS subtests, the DSST is highlighted as the most accurate predictor of brain dysfunction, and therefore its role in identifying cognitive deficits in the older adult population is integral. It is worth noting that the DSST sum score includes performance related to several abilities and can be easily affected by a low score in at least one of them [17].

Given that it is of clinical as well as statistical utility to find out which tests can detect those with SCD, the DSST, which measures processing speed and memory, should be further checked about whether it can discriminate older adults with SDC from MCI population. Namely, processing speed as well as learning and memory appear to be particularly vulnerable in older adults, deeming it rather important to focus on the adaptation of relevant neuropsychological tools in each different population. Specifically, except for processing speed and incidental learning, the test also assesses working memory, concentration, attention and visuo-perceptual functions, such as visual scanning [15]. Hence, even though a low DSST score is a non-specific one, in the sense that it lacks the ability to diagnose impairment in a single cognitive function, it should nonetheless be considered for the examinee’s general evaluation.

A typical example derives from the study by Dong, Kua, Khoo, Koo and Merchant [18] who illustrated that the DSST can be a valuable tool to detect deficits in processing speed as well as general cognitive impairment in patients with diabetes mellitus type 2, a comorbid condition in aging and also a risk factor in aMCI and Alzheimer’s disease dementia (ADD). Moreover, the DSST seems to involve visual-associative working memory as well [19], which again is impaired in early ADD. Hence, it highlights its use to differentiate MCI from early ADD population. What is also of foremost importance is that the DSST was highlighted as the cognitive test which could better predict unsafe drivers both in those without dementia as well as in early ADD stage car operators [20]. Additionally, in line with the DSST literature review in older adults, Rosano, Newman, Katz, Hirsch and Kuller [21] found that low DSST scores and low gait, separately or in combination, can be assumed as risk factors for mortality and increasing disability, independently of other risk factors. Finally, previous data [22] have shown that the DSST can be regarded as a good indicator of Activities of Daily Living (ADL) decline in patients with ADD, providing strong proof of its inevitable inclusion in the neuropsychological evaluation of this population. This finding is also illustrated by Jaeger [11], who suggests that the DSST is associated with functional disabilities in individuals with psychiatric, but also neurocognitive disorders, highlighting its use in monitoring cognitive functions in clinical practice and research.

The DSST as a basic part of the WAIS Intelligence Test versions has been repeatedly found to have satisfactory psychometric properties, and therefore is administered to distinguish populations with brain damage from their healthy counterparts. Nevertheless, to our knowledge there exist limited validation studies regarding its use in older adults, even though a majority of them undergo typical neuropsychological assessment, in order to test their cognitive performance.

The current study’s aims were formulated as follows: to examine whether the DSST can differentiate older adults with subjective cognitive decline (SCD) from those with amnestic mild cognitive impairment (aMCI), as well as those with ADD in Greek older adults’ population (2a); to examine whether it can discriminate older adults with aMCI from the population with ADD also in Greek older adults’ population (2b); and to explore whether DSST is affected by age as supported by the literature. Discriminating cognitive impairment due to ADD, MCI or SCD is very useful for neuropsychologists who work in memory clinics or similar clinical settings and conduct neuropsychological evaluations in the aforementioned populations. Therefore, this study aims at increasing knowledge about DSST literature, as well as evaluating its clinical utility in Greek population. To our knowledge, no similar study has been conducted in this population in Greece.

Based on the aforementioned literature, we expected the following:

**Hypothesis** **1** **(H1).** *The DSST would be able to discriminate among people diagnosed with SCD, aMCI and ADD*.

**Hypothesis** **2** **(H2).** *The DSST would be affected by age*.

## 2. Method

### 2.1. Procedure

The present research was a database study employing the electronic directory of the Greek Association of Alzheimer Disease and Related Disorders (Alzheimer Hellas). For the purposes of this research, three different diagnostic groups were exported: (a) community-dwelling older adults with SCD, (b) people diagnosed with aMCI and (c) patients with ADD.

#### Ethical Standards

All study participants provided their written informed consent at the time of their initial clinical visit, agreeing that the research staff of Alzheimer Hellas could use their basic demographic information such as age, gender and education, as well as their total neuropsychological test scores for research purposes. In the case of participants with ADD, due to their incapability to provide informed consent, a legal representative provided it on behalf of them.

The study was approved by the Scientific and Ethics Committee of Alzheimer Hellas (Scientific Committee Approved Meeting Number: 58-5/27-05-2020), which follows the new General Data Protection Regulation (EU) 2016/679 of the European Parliament and of the Council of 27 April 2016 on the protection of natural individuals with regard to the processing of personal data and on the free movement of such data, as well as the principles outlined in the Helsinki Declaration.

### 2.2. Participants

Out of the 1521 Greek older adults who underwent the Digit Symbol Substitution Test-WAIS-R examination from 2008 to 2019, only those with a complete medical diagnosis and a complete neuropsychological diagnosis which agreed with each other were kept in the database. People without education (illiterate) were excluded at a later stage as only very few ADD patients were found to be illiterate and this educational level was not represented in the other two diagnostic groups. Therefore, only those with low educational level (LEL), medium educational level (MEL) and high educational level (HEL) were included in the sample. As regards age, the age-range of young adults and the age range of old-old adults were not represented in all three diagnostic groups. In fact, few young adults were found only in the SCD group and few old-old adults were found only in the ADD group. Thus, participants falling into these age ranges were omitted from the study. Moreover, individuals diagnosed with moderate to severe ADD of any etiology were also eliminated. As the number of aMCI patients in the database was much larger than that of older adults with SCD and ADD, 30% of the aMCI potential participants were selected randomly (via the SPSS software command) in order to “match” the three diagnostic groups of the study in terms of the number of their participants, to some extent. The reason why we chose to randomly select 30% of aMCI patients instead of keeping all of them in the study was the small number of participants in the other two groups and especially in SCD group, and the potential consequences of number inequality on the statistical processing of the data. Therefore, because of the unequal number of participants in each group (SCD, MCI and ADD), the sample was randomized through the respective command of SPSS (in 30%). Via this method, a random sample of cases was generated. Finally, a random sample of aMCI patients and the whole populations of older adults with SCD and ADD patients of the database were used as the sample of this study, with the exceptions mentioned above.

The total study sample consisted of visitors of the two Day Care Centers (DCCs) of Alzheimer Hellas in Thessaloniki, namely, “Saint Helen” and “Saint John”, who visited the centers from 2008 to 2019 to undergo a neuropsychological and psychological routine check-up. It is worth mentioning that no participants were excluded from conducting the official evaluation, and therefore our database can be assumed as representative of Greek older adults’ population.

The diagnosis of older adults with SCD, aMCI and ADD was supported by a neurological examination, a neuropsychological and neuropsychiatric assessment, neuroimaging such as computed tomography or magnetic resonance imaging, and blood tests when possible, by consensus of specialized health professionals considered experts in neurocognitive disorders. Biomarker information, specifically APOE alleles, auditory event-related potentials (AERPs) and genetic examination (adrenoceptor alpha 2B (ADRA2B), triggering receptor expressed on myeloid cells 2 (ΤΡΕΜ2) and phospholipid-transporting ATPase (ABCA1), were also addressed in order to put each participant in the accurate diagnostic group and reassure that those who belong to the dementia group were classified as such because of Alzheimer’s disease pathology and not due to other causes. It should be noted that the participants were examined for the first time, implying that the age at the time of their diagnosis was approximately the mean age of diagnosis.

The SCD group comprised individuals who underwent an extended neuropsychological assessment at the Alzheimer Hellas DCCs as part of a yearly routine check-up. Despite being found without any objective cognitive deficits, they nonetheless reported subjective cognitive complaints and therefore could not be categorized as a healthy control sample [23,24]. Additionally, in line with previous data, there are indications that this group can be discriminated from healthy and aMCI older adults [5].

The inclusion criteria for this group were based on the SCD-I Working group criteria [25] and were formulated as follows: (a) feelings of deteriorating memory performance not associated with the presence of depressive symptoms, (b) absence of objective cognitive deficits according to the neuropsychological tests, (c) stage 2 of the disease according to Global Deterioration Scale (GDS) [26] and (d) lack of anxiety and depression according to the Geriatric Depression Scale (GDS) [27,28], the Short anxiety screening test (SAST) [29,30] and the Perceived Stress Scale [31,32]. It is worth noting that the presence of subjective complaints in this group was established prior to neuropsychological assessment during the initial history intake.

The inclusion criteria for aMCI were: (a) diagnosis of minor neurocognitive disorders according to DSM-5 [3], (b) MMSE total score 26, (c) stage 3 of the disease according to Global Deterioration Scale [26], (d) 1.5 standard deviations (SD) below average according to age and education norms in the memory domain according to the utilized neuropsychological tests and (e) absence of anxiety and depression measured by the aforementioned scales.

Finally, the inclusion criteria for ADD were: (a) diagnosis of ADD according to DSM-V criteria, (b) MMSE total score < 23, (c) stage 4 and 5 of the disease according to GDS [26] and (d) absence of anxiety and depression evaluated by the same scales employed for the two previous groups.

As for the exclusion criteria regarding all groups, they comprised: (a) history of psychiatric illness such as schizophrenia or affective disorder (major depression, generalized anxiety disorder), (b) substance abuse or alcoholism, (c) history of traumatic brain injury, (d) neurological disorders including hydrocephalus, Parkinson’s disease, encephalitis, brain tumor, epilepsy and stroke history, (e) thyroid disorders and diabetes, (g) drug treatment with opioids, B_12_, folate and (h) severe sensory deficits. It is worth mentioning that participants were evaluated by the psychiatrist of our team, to exclude clinical diagnoses of anxiety and depression, as well as history of substance use.

The neuropsychological assessment used in order to support the diagnosis included psychometric tests and specifically the Mini Mental State Examination (MMSE) [33,34] for the evaluation of general cognitive function, and the Functional Cognitive Assessment Scale (FUCAS) [35] for the evaluation of general executive function. Afterwards, neuropsychological tests were also given, in order to measure specific cognitive functions: Rey’s Verbal Learning Test (RAVLT; Greek cut-off scores from Messinis, Tsakona, Malefaki and Papathanasopoulos [36]) and the Rivermead Behavioral Memory Test (RBMT; Greek cut-off score from Efklides, Yiultsi, Kangellidou, Kounti, Dina and Tsolaki [37]) were administered to assess participants’ verbal learning as well as episodic and everyday memory, respectively. The verbal fluency test was used to measure phonemic and semantic fluency and executive functioning (Greek cut-off score from Kosmidis, Vlahou, Panagiotaki and Kiosseoglou [38]). Executive functions were also measured by the Stroop Neuropsychological Screening Test (SNST; Greek cut-off scores from Zalonis et al. [39]), as well as the Trail Making Test-part B (TMT-B; Greek cut-off scores from Zalonis et al., 2008 [40]). The Functional Rating Scale for Symptoms of Alzheimer’s Disease (FRSSD) [41] was used for evaluating ADL, while the GDS-15 ([27]; Greek cut-off score > 6 from Fountoulakis et al., [28]) and the Short Anxiety Screening Test (SAST) [29]; Greek cut-off scores from Grammatikopoulos et al. [30]) alongside the Perceived Stress Scale (PSS-14 item) [31]; Greek mean and standard deviation 25 (7.9), participants scored less than 1 SD below, from Katsarou et al. [32]) were used to exclude individuals with affective disorders. Finally, the Neuropsychiatric Inventory (NPI) [42,43] was employed for the evaluation of neuropsychiatric symptoms, and the Global Deterioration Scale to determine the stage of the diagnosis. The aforementioned neuropsychological tools were administered by licensed and trained psychologists.

### 2.3. Measures

#### The Digit Symbol Substitution Test

The Digit Symbol Substitution Test (DSST) constitutes a component of the WAIS-R battery, also standardized in the Greek population above the age of 16 [2,44]. The DSST comprises 4 series of squares. Each series is divided into 25 small empty squares, setting the total number of squares to 100. Above each square there is a number from 1 to 9. Under the numbered squares there are specific symbols, each presented under every number. During the administration, the examiner indicates the series of number–symbol pairs. The examinee is asked to draw each symbol under its corresponding number within a 90 s time limit. The total score derives from the sum of the correctly drawn symbols, implying that only one variable was taken into account during the 90 s, instead of two variables measured by other studies mentioned previously. The reason why the DSST WAIS-R was employed lies in its wide use in the Alzheimer Hellas centers for many years, and therefore a large database could be extracted for research purposes. Additionally, since the newer versions of the DSST measure primarily processing speed, which is strongly impaired with ageing, we aimed at separating the three groups by using the older version which was considered to measure learning and short-term memory. Finally, it is worth mentioning that DSST was administered after the first examination and after the diagnosis had been made.

### 2.4. Analysis

Initially, the statistical analysis was performed by the SPSS software version 25 (IBM Corp., Armonk, NY, USA). Descriptive statistics, specifically means and standard deviations, were estimated.

At the next step, in order to find the potential directed relationships of individual-demographic factors, that is, of the number of years of education, the number of years of chronological age and the gender, as well as of diagnosis (SCD, aMCI, AD dementia) with the performance in DSST, three groups of path analyses were performed separately for every two diagnostic categories (1st: for SCD and aMCI, 2nd: for SCD and ADD, 3rd: for aMCI and ADD). At this point it must be mentioned that besides age which was clearly found to correlate with digit symbol performance in the previous literature, we decided to enter education and gender variables as well, given the composition of the sample of this study. Path analysis was conducted in EQS (version 6.1) statistical software [45]. A maximum likelihood estimation procedure was performed. Regarding the confirmation of a path model, a non-significant level of goodness-of-fit index χ^2^, that is *p* > 0.05, is indicative of a good fit of the model to the data. In addition, when the value of root mean square error of approximation (RMSEA) is <0.05, it is also an indication of the good fit of the model to the data. RMSEA values ranging from 0.06 to 0.08 indicate a reasonable and therefore acceptable approximation error. Comparative fit index (CFI) examines whether the data fit a hypothesized path model compared to the basic model. Values greater than 0.90 indicate adequate fit of the model to the data, whereas values close to 1.00 indicate a good fit [46]. Moreover, to improve model fit, we examined the modification indices, namely the Wald and the Lagrange tests, which represent frequently used statistics to identify focal areas of a misfit in a path analysis solution [46].

At the last step, given the results of the path analysis, the Receiver Operating Characteristic (ROC) curve analysis was also used to assess the predictive value of DSST records to discriminate SCD from aMCI and ADD in every educational level for each age-group. At this point it must be noticed that for the needs of this type of analysis we used the categorical variables of educational level (low educational level, LEL; 2–6 schooling years, middle educational level, MEL; 7–12 schooling years, high educational level, HEL; 13≤ schooling years) and age group (50–65 years old, 66–75 years old, 76–85 years old). Finally, specificity and sensitivity were also extracted for evaluating the ability of the DSST total score to discriminate the aforementioned groups by calculating their cutoff points, which were determined by maximizing the Youden index [47]. The area under the curve (AUC) metric was used for quantifying the DSST discriminant potential in poor (0.51–0.69), fair (0.7–0.79), good (0.8–0.89), excellent (0.9–0.99) or perfect (1.0), on the basis of previous empirical evidence [48,49].

## 3. Results

Hence, the final sample comprised 488 Greek native speakers categorized in three groups: (a) people with SCD (*n* = 63, 12 men and 51 women, age range: 50 to 84 years, *M* = 67.30, *SD* = 7.07, education range: 5 to 19 years, *M* = 12.60, *SD* = 3.77), (b) people with aMCI (*n* = 182, 47 men and 135 women, age range: 50 to 85 years, *M* = 67.93, *SD* = 7.48, education range: 2 to 17 years, *M* = 12.06, *SD* = 4.10), (c) people with ADD (*n* = 243, 83 men and 160 women, age range: 53 to 86 years, *M* = 74.19, *SD* = 6.24, education range: 2 to 18 years, *M* = 8.30, *SD* = 4.34). Additionally, the sample was split into three age groups (age by decades), that is, 50–65 years old, 66–75 years old, 76–85, and into three groups based on their educational level, as measured by schooling years, that is, primary education (2–6 years; LEL), secondary education (7–12 years; MEL) and higher education (13≤ years; HEL).

The Pearson’s chi-squared analysis was used to examine gender differences between groups. The analysis indicated differences among the three groups, χ^2^ (2) = 7.044, *p* = 0.030, since the ADD group comprised more women than men compared to SCD and aMCI groups. Regarding educational level, the Pearson analysis also underlined a significant difference between groups, χ^2^ (2) = 79.532, *p* < 0.001, since people with SCD and aMCI were more educated than people with ADD. Finally, the three groups also differed in age (*p* < 0.001) since SCD and aMCI participants were younger in juxtaposition to the ADD group. In fact, older adults diagnosed with ADD were mainly women, older and less educated from older adults with SCD and aMCI. The study sample characteristics are presented in Table 1.

**Figure 1:**
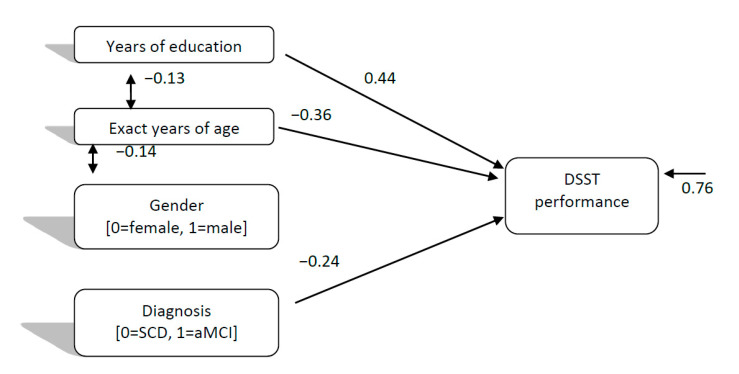
Directed relationships of individual-demographic factors and diagnosis (Subjective Cognitive Decline vs. Mild Cognitive Impairment) with digit symbol test performance.

As regards the directed relationships of individual-demographic factors and diagnosis (SCD, aMCI) with DSST performance, the path model which was finally confirmed for SCD and aMCI (Model 1), χ^2^ (5, 245) = 5.25, *p* = 0.386, CFI = 0.99, SRMR = 0.04, RMSEA = 0.01 (90%CI: 0.00–0.09), showed that diagnosis and age negatively predicted DSST performance, with younger people and people with SCD to perform better than older people and aMCI patients. However, the stronger relationship was between education and DSST performance (see Figure 1), with more educated people more likely to perform higher than people with fewer years of schooling. Thus, this model seems to confirm Hypotheses 1 and 2.

**Figure 2:**
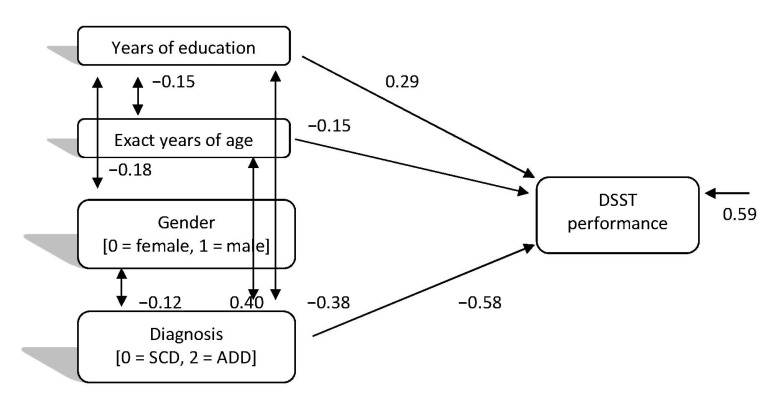
Directed relationships of individual-demographic factors and diagnosis (subjective cognitive decline vs. Alzheimer’s disease dementia) with digit symbol test performance.

In relation to the directed relationships of individual-demographic factors and diagnosis regarding SCD and ADD categories, with DSST performance, the path model which was finally confirmed (Model 2), χ^2^ (2, 306) = 0.111, *p* = 0.946, CFI = 1.00, SRMR = 0.00, RMSEA = 0.00 (90%CI: 0.00–0.01), showed more clearly the same relationships. However, as expected, the stronger relationship in this case was between diagnosis and DSST performance (see Figure 2), with people with SCD being more likely to perform well and significantly better than ADD patients. Thus, this model seems to confirm the hypotheses of the study and especially Hypothesis 1 (H1), as well. The correlations between the predictive variables in this model represent the composition of each diagnostic group and the differences between them.

**Figure 3:**
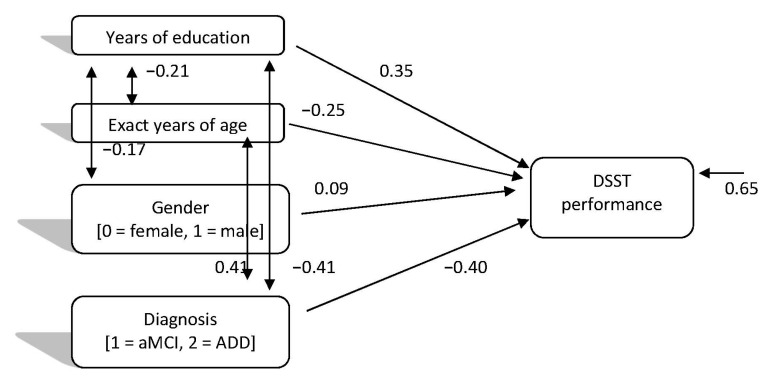
Directed relationships of individual demographic factors and diagnosis (mild cognitive impairment vs. Alzheimer’s disease dementia) with digit symbol test performance.

As for the directed relationships of individual demographic factors and diagnosis regarding aMCI and ADD categories, with DSST performance, the path model which was finally confirmed (Model 3), χ^2^ (2, 425) = 3.74, *p* = 0.153, CFI = 0.99, SRMR = 0.03, RMSEA = 0.04 (90%CI: 0.00–0.11), showed again the same relationships. In this case a small positive significant relationship was also found between gender and DSST performance, with women performing better than men. Moreover, the relationships of education and diagnosis with DSST were the stronger ones and almost of the same magnitude in the expected direction, that is, younger, more educated people and people with aMCI performed significantly better than older people with fewer years of schooling and ADD patients (see Figure 3). This model seems to confirm the hypotheses of the study too.

### DSST Discriminant Potential Regarding SCD, aMCI and ADD

The AUC of the ROC curve was used in order to quantify the DSST’s discriminant potential as fair, good, perfect or excellent. Consequently, according to the AUC, the DSST discriminant potential as regards SCD and ADD was excellent, while it was deemed good for the aMCI and mild ADD sample. However, DSST score could not detect SCD from aMCI population across all ages as well as educational groups, according to the results of our study. DSST’s AUC, cut-off scores, PPV, NPV, sensitivity and specificity scores are presented in Table 2.

## 4. Discussion

The present study aimed at providing cut-off scores for the older adult population experiencing cognitive decline, with special emphasis on the SCD, aMCI, as well as the ADD diagnostic groups. Due to the scarcity of similar studies calculating the discriminant ability of the DSST to identify the aforementioned groups, this study can be used by neuropsychologists in Greece often facing difficulties in examining older adults with cognitive impairment in clinical practice, as well as in research.

Initially, we had hypothesized that, in accordance with previous literature, age would significantly affect DSST performance. However, conducting path models including all demographic variables, that, is age, gender and education, we found that age as well as education, affected participants’ performance across the three groups of our sample. In line with this, a study measuring the German norms [50], found that demographic effects of gender, age group and educational levels can in fact be traced in the DSST scores. Moving to the education effect, path analysis showed significant educational effects on the DSST scores, corroborating the study results of Joy et al. [51] who found a small but significant main effect of educational levels on the DSST performance, as older adults with higher education outperformed their high school graduate counterparts across various age levels. Moreover, Gaertner et al. [50] suggest that education does in fact affect the DSST III performance, however, the participants’ education levels were established through a self-administered questionnaire level. As regards the role of gender, its effect on the DSST total score appeared to be trivial, as even in the study by Joy et al. [51], in which a gender effect was in fact detected, the female sample recorded only slightly increased DSST total scores in comparison to the men of the study.

By means of the discriminant potential of the DSST in terms of diagnosis, our hypothesis was partially confirmed. More specifically, this test could differentiate older adults with ADD from those with SCD as well as aMCI. However, it was not able to discriminate aMCI from SCD groups. A possible reason for this evidence lies in the fact that the cognitive functions measured by the DSST, specifically speed and memory, do not constitute skills that significantly deteriorate from the SCD to the aMCI stage and therefore no clear distinction between the older adults who belong in these groups could be found. Given that other tests such as REMEDES [5], developed to measure cognitive control abilities, the process by which goals or plans influence behavior, can distinguish patients with SCD from their aMCI counterparts, the DSST performance did not appear to be indicative of their differentiation, possibly because the tool is unable to measure cognitive control. More discussion about the main negative finding of lack of separation between SCD and aMCI in this study could include the fact that up to now in the majority of similar studies participants with SCD have been assumed to be a healthy population. Since the SCD population does not have objective impairments in their medical as well as neuropsychological records, they can be mistakenly regarded as a healthy population, whereas in reality they are not. Therefore, maybe this could be a significant reason why up to now no clear distinction has been drawn between the SCD from MCI populations. It is worth mentioning that the study of Reisberg et al. [52] highlights the fact that DSST may be predictive of progression in SCD from healthy older adult population, however, this is not contradictory to our results, because we did not recruit cognitively intact older adult participants. Moreover, this raises further questions about whether those with SCD are closer to MCI rather than cognitively older adults. Future studies could include such experiments separating healthy older adults from those with subjective cognitive complaints, and therefore enrolling them in different diagnostic categories.

On the other hand, the DSST could perfectly detect SCD patients amongst those with early ADD, which is also confirmed by previous studies. The clinical utility of the DSST in particular has been previously reported in the study by Drebing, Van Gorp, Stuck, Mitrushina and Beck [53], who supported that the DSST (WAIS-R), along with the Trail A & B tests, the RAVLT as well as the copy and recall conditions of the ROCFT constitute a compact neuropsychological battery, which can detect with high accuracy cognitive impairment in independently living older adults not diagnosed with any neurological disorders. Moreover, Joy, Kaplan and Fein [54] found that their cut-off scores can satisfactorily detect patients with ADD. Indicatively, according to Joy et al. [54], the DSST of the WAIS III was found to have satisfactory construct validity and clinical utility when assessing incidental learning. Despite its low specificity in monitoring which particular cognitive domain is impaired, the DSST represents a quite sensitive tool in identifying rather severe cognitive decline across various clinical populations [11]. Additionally, what is of foremost importance is the fact that previous studies [55] have found that patients with Parkinson’s disease outperformed those with ADD on the incidental recall adaptation performance of the DSST-WAIS-R, and therefore its use is highly recommended in assessing memory and learning functions in patients with different neurological conditions. Finally, Hoyer et al. [12] argued that the DSST test can be regarded as a valuable tool in aging research, given its close association with the tool’s measuring process, as well as perceptual speed [56].

## 5. Conclusions

Since this is the first study conducted in Greek older adults, a large gap was detected in the existing body of evidence as regards the discriminant validity of the DSST in the Greek older adult population, and its clinical utility to provide neuropsychologists with a sensitive tool to detect cognitive changes in this group. Based on our findings, it is argued that the DSST could identify cognitive deterioration as well as real-world independent living deficits in people suffering from ADD. Parallelly, no clear identification was found for the SCD population, and therefore its use is not highly recommended in neuropsychological evaluation and research. Given the existence of widely used screening tools for the detection of ADD, among others the Mini Mental State Examination, the Montreal Cognitive Assessment (MoCA; [57]) and the ADAS-cog [58], the DSST cannot be regarded as a sensitive tool to diagnose cognitive decline in the older adult population, except for ADD screening.

In summary, the current study estimated the cut-off scores of the DSST from WAIS-R available for individuals aged 50 to 84 years diagnosed with some degree of cognitive decline. Our results unequivocally support the use of the DSST in detecting patients with ADD, however, it appeared insensitive in the discrimination between SCD and aMCI populations.

## 6. Limitations

Despite the significant contribution of the current results, a number of limitations should also be outlined. Initially, no cut-off scores were estimated for the various dementia subtypes, including vascular dementia, Lewy body and frontotemporal dementia, which could have provided more prominent data regarding the decline in DSST scores among different dementia pathologies. Additionally, we did not extract cut-off scores for the specific MCI subtypes, which could provide additional insight into the aforementioned low discriminant potential of the DSST between SCD and MCI populations. Because we recruited an aMCI population, the generalizability of the DSST to discriminate MCI individuals, especially those with multiple MCI diagnoses, appears particularly dubious to establish as a definite conclusion. Moreover, a major limitation of the study is that it was done in a Greek population, so the generalizability to other populations will still be questionable. Specifically, the sample does not appear to be representative of the population from age 85<.

Furthermore, despite preexisting evidence that the DSST is associated with functional disabilities in populations with psychiatric and neurocognitive disorders, similar analyses are missing in the current study, and in this context future research could shed light on this issue in Greek older adults. Moreover, as illiterate participants were excluded from this study, the cut-off scores presented cannot be applied to populations lacking reading and writing skills. Finally, even though the IQ index was not taken into account in the current study, given that the DSST is commonly employed as auxiliary to other tests to measure intelligence, it is probable that the IQ score could impact the DSST performance. The above limitations could operate as reference points for future studies that could offer enriched knowledge regarding the qualities of this test.

## Figures and Tables

**Table 1:** Descriptive statistics for the sample’s groups.

Characteristics	SCD (*n* = 63)	aMCI (*n* = 182)	ADD (*n* = 243)	*p*
		Mean (*SD*)		
Age	67.30 (7.07)	67.93 (7.48)	74.19 (6.24)	<0.05
Gender (Male/Female)	12/51	47/135	83/160	<0.05
Years of education	12.60 (7.07)	12.06 (4.10)	8.30 (4.34)	<0.05
DSST	41.13 (12.90)	32.40 (13.25)	13.46 (9.02)	<0.05

Abbreviations: SCD: subjective cognitive decline; aMCI: Amnestic mild cognitive impairment; DSST: performance on Digit Symbol Substitution Test.

**Table 2:** Diagnostic DSST’s classification between the groups of SCD and aMCI, and aMCI and ADD stratified by age and education.

	DSST Cutoff	AUC	Sensitivity	Specificity	PPV	NPV	*p*
**SCD-MCI**							
***LEL***							
50–65	---	0.333	---	---	---	---	0.439
66–75	<24	0.761	85.7	65.2	0.59	0.92	0.039
76–85	---	0.700	---	---	---	---	0.157
***MEL***							
50–65	---	0.606	---	---	---	---	0.379
66–75	<38.5	0.825	85.7	84.1	0.67	0.97	<0.001
76–85	---	0.450	---	---	---	---	0.800
***HEL***							
50–65	---	0.651	---	---	---	---	0.141
66–75	<46.5	0.800	0.71	0.78	0.67	0.92	0.001
76–85	---	0.556	---	---	---	---	0.814
**MCI-ADD**							
***LEL***							
50–65	<19.5	0.969	0.83	1.00	0.88	1.00	0.004
66–75	<13.5	0.768	0.74	0.68	0.65	.88	<0.001
76–85	<13.5	0.820	0.75	0.87	0.61	1.00	0.034
***MEL***							
50–65	<25.5	0.922	1.00	0.80	0.77	1.00	<0.001
66–75	<24	0.836	0.75	0.81	0.73	0.93	<0.001
76–85	<21.5	0.823	70	0.82	0.65	0.99	0.002
***HEL***							
50–65	<25.5	1.000	0.97	1.00	1.00	1.00	0.005
66–75	<24	0.767	0.94	0.65	0.60	0.92	0.004
76–85	19.5	0.830	0.89	0.76	0.65	1.00	0.006
**SCD-ADD**							
***LEL***							
50–65	<20	1.000	1.00	1.00	1.00	1.00	0.014
66–75	<20	0.983	1.00	0.91	0.95	1.00	<0.001
76–85	<18	0.962	1.00	0.94	0.91	1.00	0.117
***MEL***							
***50–65***	<30	0.944	1.00	1.00	0.90	1.00	0.002
***66–75***	<27.5	0.913	0.92	0.87	0.77	1.00	<0.001
***76–85***	<19	0.890	1.00	0.71	0.75	1.00	0.026
***HEL***							
***50–65***	<33.5	1.000	0.91	1.00	1.00	1.00	0.010
***66–75***	<30.5	0.934	1.00	0.78	0.82	1.00	<0.001
***76–85***	<24	0.941	1.00	0.88	0.83	1.00	0.046

Abbreviations: DSST: Digit Symbol Substitution Test; SCD: subjective cognitive decline; MCI: mild cognitive impairment; LEL: low educational level; MEL: middle educational level; HEL: high educational level; AUC: Area under the curve; PPV: positive predictive value; NPV: negative predictive value. Note: sensitivity and specificity values are expressed in percentage.
